# Accuracy of 8 Intraocular Lens Power Calculation Formulas in Eyes Undergoing Combined Phacovitrectomy

**DOI:** 10.1155/joph/6375056

**Published:** 2026-08-27

**Authors:** Shiming Cheng, Xi Li, Qiong Yan, Jianqi Mei, Jiasheng Zhang, Guilian Shi, Junhai Lin, Tiantian Li, Ronghan Wu, A-Yong Yu

**Affiliations:** ^1^ Eye Hospital and School of Ophthalmology and Optometry, Wenzhou Medical University, Wenzhou, Zhejiang, China, wmu.edu.cn; ^2^ Department of Ophthalmology, Shenzhen Bao’an District Songgang People’s Hospital, Shenzhen, Guangdong, China; ^3^ Shanxi Eye Hospital, Shanxi Medical University, Taiyuan, Shanxi, China, sxmu.edu.cn; ^4^ National Clinical Research Center for Ocular Diseases, Wenzhou, Zhejiang, China

**Keywords:** accuracy, mean absolute error, phacovitrectomy, prediction error

## Abstract

**Purpose:**

To evaluate prediction accuracy using the 4 new‐generation formulas (Barrett Universal II (BUII), Emmetropia Verifying Optical 2.0 (EVO), Kane, and Ladas Super Formula (LSF)) and 4 traditional formulas (Haigis, Hoffer Q, Holladay 1, and SRK/T) in eyes undergoing combined phacovitrectomy.

**Methods:**

This is a retrospective case series study. In total, 249 eyes of 249 patients undergoing combined phacovitrectomy were enrolled. A single‐piece foldable monofocal IOL was implanted in each case. The patients were categorized into three subgroups according to axial length (AL) results.

**Results:**

Overall, the 4 new‐generation formulas obtained lower mean absolute prediction error (MAE) and median absolute prediction error (MedAE) of 0.42 D to 0.45 D and 0.33 D to 0.36, and higher percentage of prediction error (PE) within ± 0.50 D (65.46%–67.87%). Regarding short eyes, the Haigis formula showed the lowest MedAE of 0.34 D and the highest percentage of PE within ± 0.50 D (68.42%) with no statistical difference, and satisfactory accuracy was achieved using all formulas (all *p* > 0.05). All formulas displayed better accuracy in medium eyes with lower MAE (0.41 D to 0.46 D), lower MedAE (0.33 D to 0.37 D), and higher percentage of PE within ± 0.50 D (62.42%–69.09%) except for Hoffer Q. Both EVO and BUII formulas revealed higher accuracy in long eyes with lower MAE (0.38 D to 0.41 D), lower MedAE ranging from 0.31 D to 0.33 D, and higher percentage of PE within ± 0.50 D of both 71.74%.

**Conclusions:**

The 4 new‐generation formulas showed higher accuracy overall, and higher accuracy was achieved using EVO and BUII formulas in long eyes. All formulas revealed satisfactory accuracy in short eyes. All formulas displayed better accuracy in medium eyes except for Hoffer Q.

## 1. Introduction

With the development of surgical instruments and skills, phacovitrectomy, i.e., pars plana vitrectomy (PPV) in combination with phacoemulsification and intraocular lens (IOL) implantation, is becoming increasingly routine in many vitreoretinal conditions with coexisting cataracts. Phacovitrectomy increases the intraoperative visualization of the fundus and avoids the second surgery for cataract removal in the near future [1]. It will be important, especially for patients over 50 years old with the fast acceleration of cataracts after PPV alone due to the protein oxidation of the lens, light toxicity, side effects of tamponade materials, surgical duration, and increased oxygen tension [2–4].

Despite its advantages, combined phacovitrectomy faces some challenges. One of them is the refractive prediction accuracy. The percentage of patients with a prediction error (PE) within ± 0.5 D after cataract surgery is reported to be 73.7% based on the European Registry of Quality Outcomes in Cataract and Refractive Surgery [5]. Falkner‐Radler et al. [6] found that the concurrent phacovitrectomy group demonstrated more PE than the phacoemulsification alone group. Compared with phacoemulsification following vitrectomy, greater PE with combined phacovitrectomy was observed in a study by Tranos et al. [7]. The presence of vitreoretinal pathologies will jeopardize visual acuity to varying degrees in patients undergoing cataract surgery. Unpredictable PE will contribute to unsatisfactory visual acuity in patients undergoing combined phacovitrectomy. Thus, it is imperative to exploit the prediction accuracy of the IOL power calculation formulas in such a population.

Few studies have investigated the prediction accuracy in patients undergoing concurrent phacovitrectomy using different IOL power calculation formulas. Vounotrypidis et al. [8] probed the accuracy of 8 different formulas in 64 eyes undergoing combined phacovitrectomy with internal limiting membrane peeling. In the latest study, Hipólito‐Fernandes et al. [9] compared the accuracy of 8 formulas in 120 eyes after phacovitrectomy. Just recently, several studies have investigated the prediction accuracy in concurrent phacovitrectomy [10–12]. However, we noticed that the sample sizes are relatively small, and the conclusions are inconsistent in those studies.

Our study aimed to explore the prediction accuracy using 8 IOL power calculation formulas (4 new‐generation formulas: Barrett Universal II (BUII), Emmetropia Verifying Optical formula 2.0 (EVO), Kane, and Ladas Super Formula (LSF); 4 traditional formulas (Haigis, Hoffer Q, Holladay 1, and SRK/T) in eyes undergoing simultaneous phacovitrectomy. In addition, subgroup analysis of accuracy in short, medium, and long eyes was performed.

## 2. Methods

### 2.1. Patients

This retrospective case series included 249 eyes from 249 patients who underwent uneventful phacovitrectomy performed by the same experienced surgeon in the Fundus Surgery Department of the Eye Hospital of Wenzhou Medical University between January 2017 and May 2021. A retrospective chart review was conducted on patients who underwent concurrent phacovitrectomy. The inclusion criteria for the study were as follows: (1) concurrent uneventful phacovitrectomy; (2) in‐bag implantation of Akreos AO MI60 (Bausch & Lomb, Rochester, NY, USA), i.e., a single‐piece foldable monofocal IOL; (3) age greater than 18 years; (4) postoperative corrected distance visual acuity (CDVA) ≥ 20/40; (5) follow‐up duration of at least 3 months. The exclusion criteria were as follows: (1) keratopathy, lens dislocation, glaucoma, uveitis, or ocular trauma; (2) a previous history of corneal refractive surgery, scleral buckling surgery, or posterior scleral reinforcement surgery; (3) preoperative retinal detachment and/or choroidal detachment; (4) axial length (AL) values obtained via A‐scan; (5) postoperatively silicone oil tamponade; and (6) intraoperative or postoperative complications such as choroidal hemorrhage or endophthalmitis. All patients provided written informed consent prior to surgery. If both eyes of a patient met the eligibility criteria, only the eye with better CDVA was enrolled. This study was approved by the Ethics Committee of the Eye Hospital of Wenzhou Medical University (approval number 2021–144‐K‐123). Due to the retrospective design of the study, informed consents from the patients were waived under this approval. All procedures in this study were conducted in accordance with the Declaration of Helsinki.

### 2.2. Biometry and IOL Power Calculation

The IOL‐Master (V5.4, Carl Zeiss Meditech, Jena, Germany) was utilized to obtain preoperative biometric measurements for IOL. These procedures were conducted by trained professionals at the Clinical Examination Center. In cases where AL measurements could not be obtained using the IOL‐Master, the OA‐2000 (Tomey, Nagoya, Japan) was employed. Subjective refraction outcomes were extracted at least 3 months postoperatively, and spherical equivalents (SE) were subsequently calculated.

PE was defined as the difference between postoperative SE and each formula‐predicted SE based on the actual power of implanted IOL. The study included eight IOL power calculation formulas: four traditional formulas—Haigis, Hoffer Q, Holladay 1, and SRK/T and four new‐generation formulas‐BUII, EVO, Kane, and LSF (a. The BUII formula. Last accessed January 8, 2022. http://calc.apacrs.org/barrett_universal2105/; b. The EVO 2.0 formula. Last accessed January 8, 2022. https://www.evoiolcalculator.com/calculator.aspx/; c. The Kane formula. Last accessed January 10, 2022. https://www.iolformula.com/; and d. The LSF. Last accessed January 10, 2022. https://www.iolcalc.com/). Using an A constant of 119.1, the formula‐predicted SE of each new‐generation formula was obtained via online data entry of keratometry, anterior chamber depth (from the corneal epithelium to the anterior surface of the lens, ACD), and AL on the respective websites a, b, c, d. Given the accessibility, we opted not to perform lens constant optimization for the four new‐generation formulas. According to the User Group for Laser Interference Biometry (ULIB), the lens constants for the MI 60 were as follows: A constant (SRK/T) = 119.1, pACD (Hoffer Q) = 5.67, SF (Holladay 1) = 1.90, a0 = 1.49, a1 = 0.40, and a2 = 0.10 (lens constants for Haigis). Based on the original publications and errata, the Haigis [13], Hoffer Q [14, 15], Holladay 1 [16], and SRK/T [17] formulas were programmed into Excel 2016 (Microsoft, Washington, USA) by J.Q.M. Data from 100 patients were utilized to calculate the optimized lens constants for the four traditional formulas using the “Goal Seek” function in Excel 2016, as proposed by Hoffer and Savini [18]. Following optimization, the lens constants for the MI 60 were as follows: A constant (SRK/T) = 119.046, pACD (Hoffer Q) = 5.857, SF (Holladay 1) = 1.989, a0 = 1.614, a1 = 0.40, and a2 = 0.10 (three A constants for Haigis). In our study, the a0 constant for the Haigis formula was optimized, while the values for a1 and a2 were obtained from the ULIB website for the IOL‐Master, consistent with the methodology employed by Melles et al. [19]. The optimized lens constants were utilized to calculate the formula‐predicted SE, after which the PE was computed for each of the four traditional formulas.

### 2.3. Surgery Process

After the insertion of three 23‐gauge trocars, a superior temporal 2.2‐mm clear corneal incision was made, followed by a continuous curvilinear capsulorrhexis of approximately 5.5 mm, to facilitate standard phacoemulsification and the implantation of a MI60 IOL into the capsular bag. Subsequently, vitrectomy was performed using standard techniques. For patients with macular holes, peeled internal limiting membranes were employed for macular holes tamponade. A circular 3.5‐mm posterior capsulotomy was performed for each patient to prevent posterior capsule opacification. Perfusion fluid, disinfected air, or C3F8 was administered to fill the vitreous chamber, depending on the vitreoretinal disease and specific intraoperative conditions.

### 2.4. Groupings

According to the method from Melles et al. [19], the patients were categorized into three groups: short eyes (AL ≤ 22.50 mm), medium eyes (> 22.50 mm to < 25.50 mm), and long eyes (AL ≥ 25.50 mm).

## 3. Statistical Analysis

Statistical analyses were performed using SPSS (Version 25, Chicago, USA) and Excel 2016. A one‐sample *t*‐test or Wilcoxon signed‐rank test was applied to evaluate whether the PE of each IOL power calculation formula differed significantly from 0. Mean numerical PE (ME) and mean absolute PE (MAE) were presented as the mean ± standard deviation. One‐way analysis of variance was used to assess PE between different formulas as suggested [18]. The Friedman test was applied to detect MAE/median absolute PE (MedAE) differences among the eight formulas followed by Wilcoxon signed‐rank post hoc tests with Bonferroni’s correction for multiple pairwise comparisons in case of significance: *p* < 0.05. Comparisons of the percentage of PE within ± 0.25 D, ± 0.50 D, ± 0.75 D, and ± 1.00 D among different formulas were conducted using the Cochran Q test. Adjusted *p* value < 0.05 indicates statistical significance. The prediction accuracy was evaluated based on MAE, MedAE, and the percentage of PE within ± 0.50 D. According to the study by Stopyra et al., at least 110 eyes were needed to obtain a confidence level of 95% [20].

## 4. Results

### 4.1. Baseline Characteristics and Demographics

A total of 249 eyes from 249 patients were enrolled in the study. The average age at surgery was 65.04 ± 9.19 years (range: 25–83 years). Among all subjects, the percentage of females was 69.08%, while that of males was 30.92%. The study included 127 right eyes and 122 left eyes. The mean preoperative CDVA was 0.73 ± 0.66 logMAR; the mean postoperative CDVA was 0.15 ± 0.19 logMAR. Follow‐up duration ranged from 3 to 56 months with a mean of 10.47 ± 9.12. Detailed ocular parameters are presented in Table 1. Out of the 249 eyes, AL outcomes of 12 eyes were acquired with the OA‐2000, which could not be measured with the IOL‐Master.

**TABLE 1:** Preoperative biometric characteristics of overall and axial length subgroups.

Parameters	Overall	Short eyes	Medium eyes	Long eyes
No. of eyes	249	38	165	46
Keratometry (D)	44.47 ± 1.43	45.85 ± 1.15	44.18 ± 1.28	44.36 ± 1.55
Mean ± SD/range	(41.09, 48.46)	(42.89, 48.46)	(41.09, 47.12)	(41.13, 48.01)
ACD (mm)	3.13 ± 0.41	2.77 ± 0.28	3.10 ± 0.34	3.55 ± 0.37
Mean ± SD/range	(1.97, 4.24)	(2.21, 3.52)	(1.97, 3.92)	(2.43, 4.24)
Axial length (mm)	23.33 (22.77, 24.66)	22.23 (21.93, 22.43)	23.28 (22.95, 24.11)	28.77 ± 1.89^#^
Median (interquartile)/range	(20.91, 33.84)	(20.91, 22.50)	(22.51, 25.49)	(22.58, 33.84)
IOL powers (D)	21.50 (18.00, 23.00)	23.75 (23.00, 25.00)	21.50 (20.00, 22.50)	9.51 ± 4.38^#^
Median (interquartile)/range	(2.00, 28.50)	(21.00, 28.50)	(13.50, 25.50)	(2.00, 18.00)

Abbreviation: ACD, anterior chamber depth; SD, standard deviation; D, diopters.

The indications for PPV included epiretinal membranes (ERMs, 134 eyes), macular holes (58 eyes), proliferative diabetic retinopathy (PDR, 26 eyes), vitreous opacities related to pathological myopia (14 eyes), vitreous hemorrhage secondary to branch retinal vein occlusion or polypoidal choroidal vasculopathy (10 eyes), vitreomacular traction (5 eyes), and retinoschisis (2 eyes).

### 4.2. Comparison of Prediction Accuracy of IOL Power Calculation Formulas in all Patients

**FIGURE 1:**
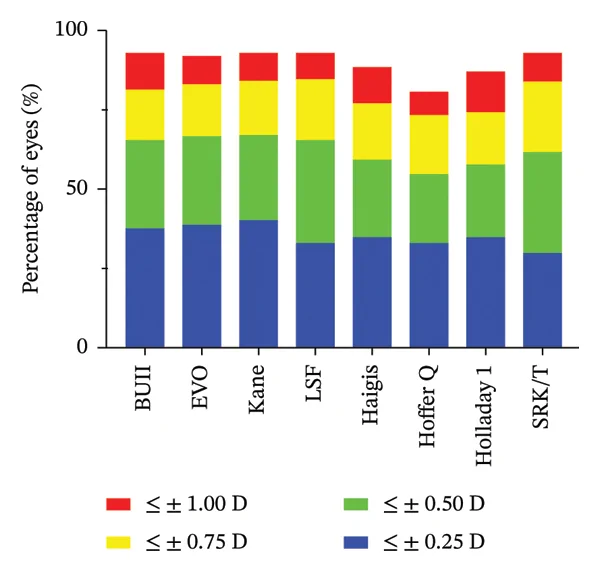
The stacked graph of the percentage of prediction error ±0.250, ±0.50, ±0.75, and ±1.00 diopters (D) using the eight formulas overall.

Table 2 and Figure 1 present the overall prediction accuracy results for the eight IOL power calculation formulas. PE did not significantly differ from zero across all eight formulas (all *p* > 0.05). No statistically significant differences were observed among the eight formulas regarding PE (*p* = 0.79). In terms of MAE and MedAE, the Kane formula represented the lowest values; significant pairwise differences were found between the Hoffer Q and either of the 4 new generation formulas, between Kane and Haigis formulas, between Kane and Holladay 1 formulas, and between Kane and SRK/T formulas (*p* < 0.05).

**TABLE 2:** Overall prediction accuracy outcomes for 8 IOL calculation formulas.

Parameters	Kane	EVO	BU II	LSF	SRK/T	Haigis	Holladay 1	Hoffer	*p*
ME (D)	−0.07 ± 0.54	−0.04 ± 0.55	0.00 ± 0.56	0.00 ± 0.56	0.01 ± 0.58	−0.04 ± 0.62	−0.04 ± 0.66	−0.06 ± 0.75	0.792
MAE (D)	0.42 ± 0.34	0.43 ± 0.35	0.44 ± 0.34	0.45 ± 0.34	0.46 ± 0.35	0.49 ± 0.38	0.51 ± 0.42	0.58 ± 0.49	< 0.001^∗^
MedAE (D)	0.33	0.34	0.34	0.36	0.39	0.40	0.44	0.43	< 0.001^∗^
± 0.25 D (%)	39.76	38.96	38.15	32.93	29.72	34.54	34.94	32.93	0.017^∗^
± 0.50 D (%)	67.87	66.67	65.46	65.86	62.25	59.84	57.83	55.02	< 0.001^∗^
± 0.75 D (%)	84.74	84.74	81.93	84.74	84.34	77.91	74.30	73.49	< 0.001^∗^
± 1.00 D (%)	93.98	93.98	93.98	93.98	93.57	88.76	87.15	81.53	< 0.001^∗^

*Note:* EVO, Emmetropia Verifying Optical formula Version 2.0; LSF, Ladas Super Formula; prediction error; ME, mean numerical prediction error; MedAE, median absolute error; ± 0.25 D (%), ± 0.50 D (%), ± 0.75 D (%), ± 1.00 D (%), percentage of prediction error within ± 0.25 D, ± 0.50 D, ± 0.75 D, and ± 1.00 D, respectively.

Abbreviation: BUII, Barrett Universal II; D, diopters; MAE, mean absolute error.

^∗^Statistically significant difference (*p* < 0.05).

Significant difference was only found between Kane (39.76%) and SRK/T (29.72%) with regard to percentage of PE within ± 0.25 D (*p* < 0.05). In terms of percentage of PE within ± 0.50 D, the Hoffer Q formula exhibited lower values than any of the four new‐generation formulas (*p* < 0.05); the SRK/T formula showed lower values than the Kane formula (*p* < 0.05).

The 4 new‐generation formulas displayed better overall prediction accuracy with regard to MAE, MedAE, and the highest percentage of PE within ± 0.50 D.

### 4.3. Subgroup Analyses of Prediction Accuracy of IOL Power Calculation Formulas

**FIGURE 2:**
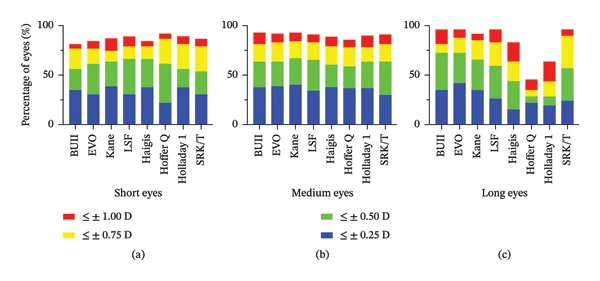
The stacked graph of the percentage of prediction error ±0.250, ±0.50, ±0.75, and ±1.00 diopters (D) using the eight formulas in subgroups.

The accuracy outcomes of the eight IOL power calculation formulas in short eyes are shown in Table 3 and Figure 2a. The differences among the 8 formulas for MAE and MedAE were not statistically significant based on the pairwise comparisons (all *p* > 0.05). No statistically significant differences were observed among the eight formulas in terms of the percentages of PE within ± 0.25 D and ± 0.50 D based on pairwise comparisons (all *p* > 0.05). All 8 formulas revealed satisfactory accuracy results in short eyes with no significant difference.

**TABLE 3:** Prediction accuracy outcomes in subgroups of axial length.

	Kane	EVO	BUII	LSF	SRK/T	Haigis	Holladay 1	Hoffer	*p*
Short eyes									
MAE	0.47 ± 0.41	0.49 ± 0.41	0.52 ± 0.43	0.47 ± 0.39	0.51 ± 0.41	0.46 ± 0.40	0.44 ± 0.39	0.47 ± 0.37	0.124
MedAE	0.40	0.40	0.34	0.34	0.42	0.34	0.45	0.36	0.124
± 0.25 D	39.47	31.58	36.84	31.58	31.58	39.47	39.47	26.31	0.364
± 0.50 D	65.79	63.16	57.89	68.42	55.26	68.42	57.89	63.16	0.540
± 0.75 D	76.32	78.95	78.95	81.58	81.58	81.58	84.21	89.47	0.217
± 1.00 D	89.47	86.84	84.21	92.11	89.47	86.84	92.11	94.74	0.162
Medium eyes									
MAE	0.41 ± 0.33	0.43 ± 0.35	0.43 ± 0.33	0.44 ± 0.34	0.44 ± 0.35	0.46 ± 0.38	0.44 ± 0.38	0.49 ± 0.40	0.001^∗^
MedAE	0.33	0.34	0.35	0.33	0.37	0.37	0.36	0.39	0.001^∗^
± 0.25 D	41.21	40.00	39.39	35.15	30.91	38.79	38.18	38.18	0.126
± 0.50 D	69.09	66.06	65.45	67.27	65.45	62.42	66.06	60.61	0.142
± 0.75 D	86.67	85.45	83.03	86.06	83.64	81.21	80.61	80.61	0.059
± 1.00 D	95.76	95.15	95.76	93.94	93.94	90.91	92.73	88.48	< 0.001^∗^
Long eyes									
MAE	0.44 ± 0.33	0.38 ± 0.31	0.41 ± 0.31	0.47 ± 0.30	0.48 ± 0.28	0.63 ± 0.36	0.80 ± 0.47	0.99 ± 0.62	< 0.001^∗^
MedAE	0.35	0.31	0.33	0.46	0.42	0.61	0.86	1.13	< 0.001^∗^
± 0.25 D	34.78	41.30	34.78	26.09	23.91	15.22	19.57	21.74	0.052
± 0.50 D	65.22	71.74	71.74	58.70	56.52	43.48	28.26	28.26	< 0.001^∗^
± 0.75 D	84.78	86.96	80.43	82.61	89.13	63.04	43.48	34.78	< 0.001^∗^
± 1.00 D	91.30	95.65	95.65	95.65	95.65	82.61	63.04	45.65	< 0.001^∗^

*Note:* EVO, Emmetropia Verifying Optical formula Version 2.0; LSF, Ladas Super Formula; prediction error; ME, mean numerical prediction error; MedAE, median absolute error; ± 0.25 D (%), ± 0.50 D (%), ± 0.75 D (%), ± 1.00 D (%), percentage of prediction error within ± 0.25 D, ± 0.50 D, ± 0.75 D, and ± 1.00 D, respectively.

Abbreviation: BUII, Barrett Universal II; D, diopters; MAE, mean absolute error.

^∗^Statistically significant difference (*p* < 0.05).

The accuracy outcomes of the eight formulas in medium eyes are presented in Table 3 and Figure 2b. Statistical difference was observed only between the Kane and Hoffer Q formulas (*p* < 0.05) for MAE and MedAE. No significant difference was detected among the eight formulas for percentage of PE within ± 0.25 D and ± 0.50 D (all *p* > 0.05).

Table 3 and Figure 2c present the accuracy outcomes of eight formulas in long eyes. The four new‐generation formulas and SRK/T detected lower MAE and MedAE than Hoffer Q (*p* < 0.05) and both EVO and BUII formulas exhibited lower MAE and MedAE values than Holladay 1 (*p* < 0.05). Regarding percentage of PE within ± 0.25 D, no significant differences were detected among the eight formulas. As for percentage of PE within ± 0.50 D, the four new‐generation formulas exhibited higher values than Hoffer Q and Holladay 1 (*p* < 0.05). The SRK/T formula displayed higher percentage of PE within ± 0.50 D than Hoffer Q and Holladay 1 (*p* < 0.05); both EVO and BUII formulas exhibited higher values than Haigis (*p* < 0.05). Satisfactory accuracy results were achieved with EVO and BUII formulas in long eyes.

### 4.4. Correlations of Each Formula With AL

Pearson correlation test was adopted to assess whether a correlation existed between AL and absolute error (AE) of each IOL power calculation formula. There were positive correlations between AL and AE of the Kane, LSF, Haigis, Hoffer Q, Holladay 1, and SRK/T formulas (*p* < 0.05). No significant correlation between AL and AE was observed using the EVO and Barrett UII formulas (*p* > 0.05).

## 5. Discussion

Our findings demonstrated that the four new‐generation formulas exhibited higher prediction accuracy overall in eyes after phacovitrectomy. In short eyes, good prediction accuracy was detected with all 8 formulas. As for medium eyes, higher prediction accuracy was accessed with all formulas except for Hoffer Q. In the subgroup of long eyes, higher accuracy was achieved using EVO and BU II formulas.

Previous studies have reported a myopic shift in eyes undergoing combined phacovitrectomy, ranging from −0.02 to −0.40D [6, 21–23]. Several factors have been reported to be associated with this phenomenon, including poor preoperative visual acuity, foveal or retinal detachment, increased central macular thickness, shallow ACD, and long AL [7, 24]. In contrast, other previously published studies have not observed a myopic shift in eyes undergoing combined phacovitrectomy [12, 25, 26]. Similarly, our study did not detect a significant myopic shift when employing four new‐generation formulas. This discrepancy may be attributed to the heterogeneous nature of retinal pathologies and variability among surgeons [10].

Overall, the four new‐generation formulas exhibited higher prediction accuracy overall in eyes after phacovitrectomy in our study; the Kane formula achieved the highest prediction accuracy with no significant differences among the four new‐generation formulas. Consistent with our results, Vounotrypidis et al. [8] reported that the Kane formula achieved the best accuracy in eyes undergoing combined phacovitrectomy. Similarly, in a study by Hipólito‐Fernandes et al. [9], the Kane formula exhibited better accuracy than other formulas, including PEARL‐DGS and EVO in eyes undergoing combined phacovitrectomy. The Kane and BUII formulas demonstrated superiority over other IOL power calculations formulas in phacovitrectomy in the study by Thanitcul et al. [10]. Sato et al. [12] detected that the highest prediction accuracy was obtained with the BU II formula. Based on the aforementioned outcomes, both the Kane and BUII formulas are preferable for use in phacovitrectomy.

In general, satisfying accuracy results were obtained with all formulas in short eyes in the current study. A recently published study on accuracy in short eyes (14 eyes) after phacovitrectomy showed that the new‐generation formulas, including the BU II, EVO, Kane, and LSF, surmounted Holladay 2 and 4 traditional formulas with lower MAE and MedAE [12]. With regard to medium eyes, higher accuracy was obtained with all formulas except for Hoffer Q with the highest accuracy obtained with the Kane formula (no significant differences among 7 formulas) in the present study. The study by Sato et al. [12] reported that the BU II formula achieved the highest accuracy in medium eyes after concurrent phacovitrectomy. Unfortunately, subgroup analysis of medium eyes was not conducted in the study by Vounotrypidis et al. [8] and Hipólito‐Fernandes et al. [9] in eyes undergoing combined phacovitrectomy.

As for long eyes, the EVO and BU II formulas demonstrated better accuracy in our study. The Haigis and BUII formulas acquired lower MAE among the 9 IOL formulas in long eyes after phacovitrectomy in the study by Sato et al. [12]. Furthermore, Tanaka et al. [27] found that the SRK‐T and BUII formulas demonstrated superior accuracy compared to the Kane and Hill‐RBF formulas in long eyes (> 26 mm) following phacovitrectomy for epiretinal membrane. However, it is important to note that only eight long eyes were included in that study. Variability in studies examining accuracy in phacovitrectomy may have led to inconsistencies and divergent conclusions. This variability may be attributed to the heterogeneity of subject cohorts, racial diversity, formulas used, biometric devices, sample sizes, and surgeons involved. To date, research on the accuracy of eyes following phacovitrectomy remains limited. Further related studies are anticipated in the future.

Pearson correlation analysis was employed to investigate the relationship between AL and AE in the current study. The results indicate that the correlation was observed in all formulas except for the EVO and BUII formulas. The AL was not correlated with AE in the EVO and BUII formulas in the present study. This finding elucidates why the EVO and BUII formulas demonstrated superior prediction accuracy compared to other formulas in long eyes. Consequently, the EVO and BUII formulas are recommended for use in long eyes undergoing combined phacovitrectomy.

### 5.1. Limitation

However, several limitations exist in this study. First, the study was limited by its retrospective nature. Further prospective clinical trials are required to validate our findings. Second, the absence of lens thickness and white‐to‐white measurements precluded the application of certain formulas, including Holladay 2, Olsen‐4, and Pearl DGS. Third, the study utilized a single‐model IOL, and further investigation is warranted to determine whether the conclusions can be generalized to other IOL models. Fourth, due to the difficulty of obtaining lens constant optimization for the new‐generation formulas and the potential costs involved, lens constant optimization was not performed for the 4 new‐generation formulas. It may result in conclusion bias to certain extent. Fifth, small sample sizes in the short and long eye subgroups may also contribute to certain conclusion bias. Sixth, diversity of retinal pathologies may also exert an influence on refractive error. Seventh, OA‐2000 was used for biometry in 12 eyes with the exception of 237 eyes with the IOL‐Master. Eighth, root‐mean‐square absolute error (RMSAE), which is a currently recommended index, was not applied in our study.

## 6. Conclusion

In conclusion, the four new‐generation formulas demonstrated better prediction accuracy overall in eyes undergoing combined phacovitrectomy. Generally, all formulas obtained satisfactory prediction accuracy in short eyes. In medium eyes, all formulas demonstrated better prediction accuracy except for Hoffer Q. Higher prediction accuracy was exhibited employing both EVO and BU II formulas in long eyes. Further studies with large sample size and high quality are urgently needed to explore prediction accuracy in eyes underwent phacovitrectomy with more recently established formulas.

## Author Contributions

Conceptualization: Shiming Cheng; methodology: Shiming Cheng and A‐Yong Yu; data collection and analysis: Shiming Cheng, Xi Li, Qiong Yan, Jianqi Mei, Jiasheng Zhang, Guilian Shi, Junhai Lin, and Tiantian Li; writing–original draft: Shiming Cheng and A‐Yong Yu; writing–review and editing: Shiming Cheng, Xi Li, Qiong Yan, Jianqi Mei, Jiasheng Zhang, Guilian Shi, Junhai Lin, Tiantian Li, Ronghan Wu, and A‐Yong Yu; supervision: Ronghan Wu and A‐Yong Yu; funding acquisition: A‐Yong Yu.

## Funding

This study was funded by the Wenzhou Key Team of Scientific and Technological Innovation (Grant number C20170002), Wenzhou Public Welfare Science and Technology Projects (Grant number Y20170192), Young Talents Programme of Zhejiang Medical and Health Science and Technology Project (Grant number 2019RC223), Engineering Development Project of Ophthalmology and Optometry (Grant number GCKF201601), Nature and Science Foundation of China (Grant number 81570869).

## Disclosure

No financial disclosures. The funding organization had no role in the design or conduct of this research.

## Conflicts of Interest

The authors declare no conflicts of interest.

## Data Availability

The data that support the finds of this study are available on proper request from the corresponding author. The data are not publicly available due to privacy or ethical restrictions.
